# Supplementary material for: Studying Venom Toxin Variation Using Accurate Masses from Liquid Chromatography–Mass Spectrometry Coupled with Bioinformatic Tools
Source: Toxins (Basel). 2024 Apr 7;16(4):181. doi: 10.3390/toxins16040181 (PMC11053424; doi:10.3390/toxins16040181)
Supplement: Supplementary file 1 [file toxins-16-00181-s001.zip › Supplementary Material.pdf]

Supplementary Materials

Studying Venom Toxin Variation Using Accurate Masses from Liquid Chromatography–Mass Spectrometry Coupled with Bioinformatic Tools

Luis L. Alonso, Jory van Thiel, Julien Slagboom, Nathan Dunstan, Cassandra M. Modahl, Timothy N. W. Jackson, Saer Samanipour and Jeroen Kool

Contents

S1. Detailed comparison between manually and automatically extracted data 2

S2. PCs and loadings of independent genera 4

S3. HT venomics materials and methods 7

S3.1 Processing of High Throughput venomics data 9

S1. Detailed comparison between manually and automatically extracted data

The first comparison thresholds were set as follows: if the toxins were similar both in mass (±2Da) and *m*/*z*-value (±1.1) they were considered as the same toxin if they were found in the same venom. This led to 41.9% of the toxin sample pairs and 57.3% of the unique toxins matching. The relationship between the peak heights obtained manually and the areas obtained automatically were studied to recognize possible errors. The results showed that this relationship was linear (as expected because such is the relationship in gaussian peaks [27]), except for 2.9% of the results (Confidence Interval -CI-, 95%). This small percentage of toxin sample pairs would not match due to an under-estimation of the peak height when they were manually deconvoluted (i.e., the manual extraction had some errors, which the automatic extraction was able to surpass). However, this is not an accurate recognition method, as we know the manual deconvolution can render different results from the automatic one due to human error or the automatic method calculating the accurate mass from the isotopic average of the atoms that conform the toxin. For example, two toxins can be given the same most abundant *m*/*z*-value but, when the deconvolution process is different, gives different masses (e.g., 7907.73 Da vs 7905.75 Da). To solve this issue, the same threshold was set for both Mass (±2Da) and for *m*/*z*-value (±1.1), but all toxins within the same venom that fall in either of these two conditions were considered to be the same toxin. After this comparison was performed, 74.2% of the toxin sample pairs were recognized as being the same between both extraction methods, and 83.7% of the extracted toxins matched. The relationship between the intensities obtained manually and the areas obtained automatically was studied again to recognize any deviations in the linear trend that relates these parameters. If the relationship between both variables remains constant, then that would indicate that the addition of this new threshold is coherent with the previous results, just enhancing the number of matches found. The results showed that this relationship was indeed

constant, even reducing the standard error, meaning that it not only supports the previous results, but in this case, 3.6% of the peak heights (CI, 95%) were underestimated. Also, 0.6% of the areas were overestimated, an issue that occurred due to the program recognizing a same toxin several times but giving it a different *m*/*z*-value each time it was recognized.

When comparing the errors found in both the intercept and the slope of the three methods used to compare the data, it was found that the standard error decreased both by going from method 1 to method 2, and from method 2 to method 3, whereas the correlation between the peak height of the manually deconvoluted toxins and the area of the automatically deconvoluted ones was maintained. This indicates that the robustness obtained using the first method (i.e., toxin parameters needing to match both in *m*/*z*-value and mass) was maintained when using the third method. The relative errors of the intercept and slope of the correlation between peak height vs peak area decreased from 24 to 2% and from 10 to 6% respectively throughout methods 1 to 3. Thus, this proves that the results from the automatic deconvolution render very comparable results to those of the manual extraction. Data obtained through the automatic extraction and deconvolution was now analysed using PCA in the same manner done by van Thiel *et al* [10]. This allowed for a comparison of the results obtained by PCA dimensionality reduction techniques between the two methods, to test whether similar PCA results were achieved.

S2. PCs and loadings of independent genera

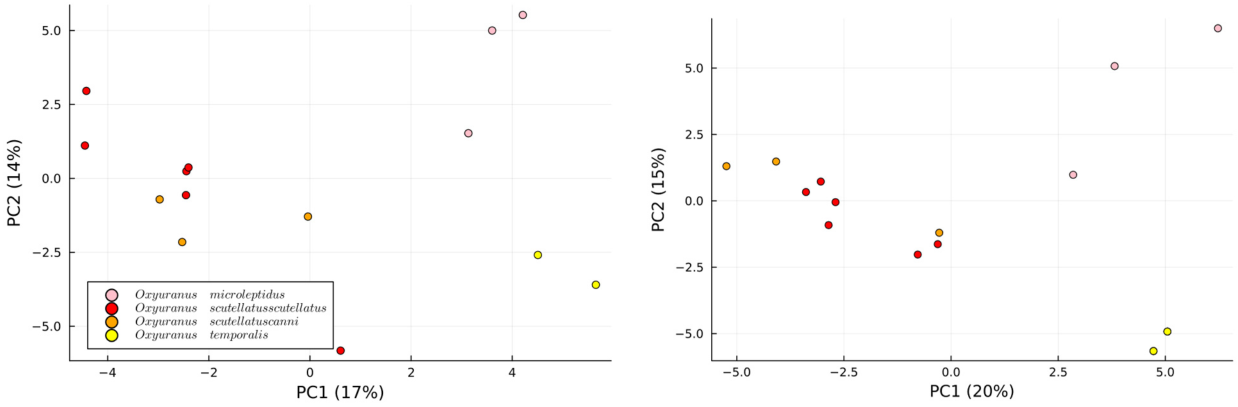

Supplementary Figure S1. PCA representation of the manually extracted data (left) and automatically extracted data (right) for the Oxyuranus samples. In both cases there is a clear difference between the three species, but in the automatically processed data, PC2 also reveals that one of the Oxyuranus scutellatus samples is different from the rest, this is the only one coming from the norther territory.

The *Oxyuranus* plots shows strong similarity between the manual and automatic processed data (Supplementary Figure 1). The clustering pattern between *Oxyuranus* species is generally similar, with only minor differences observed within *O. scutellatus* venoms (Supplementary Figure 1). The first two dimensions of the PCA clearly differentiate the three taipan species.

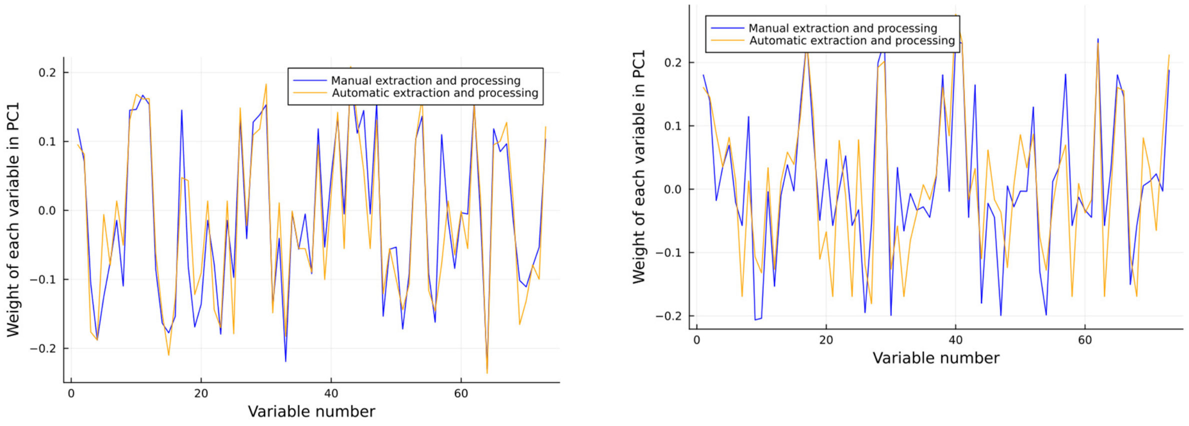

Supplementary Figure S2. Representation of the loadings of the PCAs 1 and 2 of the manually extracted data (blue) and automatically extracted data (orange) for the Oxyuranus samples PCAs. In both cases, the variables with the highest weights follow the same trend.

By plotting the PCA loadings (i.e. the matrix containing the relevance of each variable for each PC-plot) in Supplementary Figure 2, it was found that this differentiation is generated due to the presence of certain toxins. Three responsible masses were identified in *O. microlepidotus* (i.e. 13327.8 Da, 13365.8 Da and 13853.1 Da) and *O. temporalis* (i.e. 6697.9 Da, 7718.6 Da and 13326.9 Da), and some masses that were found in both of these species but not in *O. scutellatus* (i.e. 13385.7 Da and 6704.9 Da). Using the automatic approach, we observed one specific venom that deviated relatively more within the *O. scutellatus* venoms (Supplementary Figure 1), as it can be found at the lowest values of the 2nd PC. This is the only venom within our dataset originating from an isolated *O. scutellatus* population in Northern Territory. The toxins that were unique for this venom and thereby contributed to the relative deviation in the *O. scutellatus* are the ones with masses of 7905.7 Da, 14182.0 Da and 13225.8 Da).

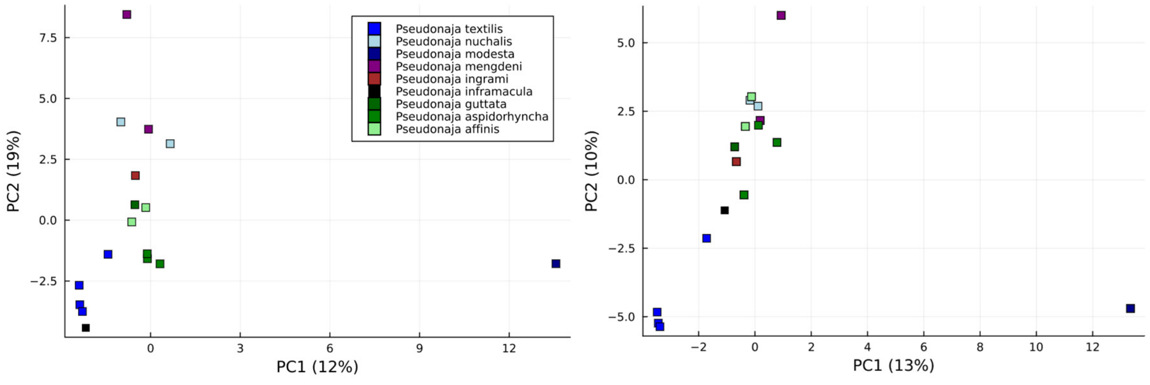

Supplementary Figure S3. PCA representation of the manually extracted data (left) and automatically extracted data (right) for the Pseudonaja samples. P. modesta is a clear outlier in PC1, whereas P. testilis is also highly differentiated by PC2.

The same analysis on all *Pseudonaja* venoms show a similar clustering pattern between manual and automatic PCAs (Supplementary Figure 3). When the two dimensions of the PCA for the *Pseudonaja* dataset are considered, *P. modesta* is a venom that differentiates itself completely from the rest of the *Pseudonaja*. This is mainly due to the lack of toxins that are found in the other venoms, and the presence of 3 specific toxins in high quantities: 6706.2, 14305.0, 13478.7 Da. Most interestingly, previous studies also suggest that the lack of high molecular weight toxins, such as venom factor X and V, are responsible for their unique venom phenotype among *Pseudonaja* species [11,39]. Regarding the other *Pseudonaja* species, it is interesting to note how *P. mengdeni* and *P. nuchalis* cluster together, which is primarily due to several toxins only shared by these two species (e.g.: 3858.0 and 3425.8 Da) and toxins that are present in other venoms but at much lower levels (e.g.: 6838.8, 7392.3, and 14267.9 Da). Also, *P. textilis* and *P. ingrami* venoms tightly cluster together, which is mainly due to toxins that are found more abundantly in these venoms (e.g.: 6490.9 and 6680.9), and toxin 6827.8, the last also found in *P. aspidorhyncha* and *P. affini* venoms. In Supplementary Figure 4 it is shown that their loadings also follow the same pattern.

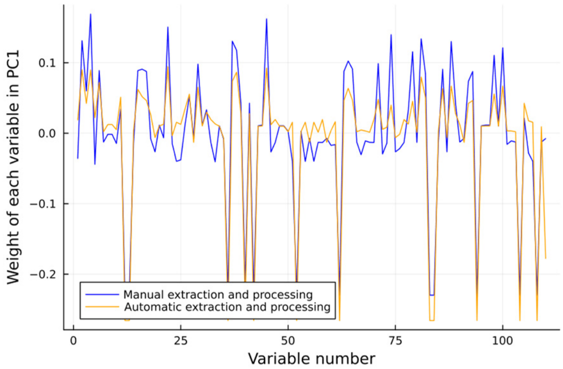

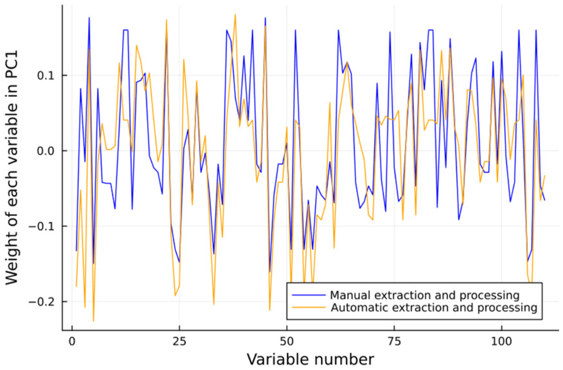

Supplementary Figure S4. Representation of the loadings of the PCAs 1 and 2 of the manually extracted data (blue) and automatically extracted data (orange) for the Pseudonaja samples PCAs. In both cases, the variables with the highest weights follow the same trend.

S3. HT venomics materials and methods

For HT venomics, the venoms from the taipan snakes (*Oxyuranus*) were pooled and also the venoms from the brown snakes (*Pseudonaja*) were pooled. These two venom samples were separated and analysed using LC-MS as described by van Thiel *et al* [10]. Additionally, after the post-column split, the larger portion of the eluent (i.e., 90%) was transferred to a FractioMateTM FRM100 nanofraction collector (SPARK-Holland & VU Amsterdam, Emmen & Amsterdam, The Netherlands). The FractioMate was controlled by FractioMator software. LC-fractions (one every 6 seconds) were collected on transparent 384-well plates in a serpentine fractionation pattern. Afterwards, the 384-well plates were vacuum-centrifuged overnight for 16 h using a Christ Rotational Vacuum Concentrator RVC 2-33 CD plus (Salm en Kipp, Breukelen, The Netherlands). The plates were then stored at –20 °C until further experimental use.

For the subsequent tryptic digestion step, 25 µL of reduction buffer (25 mM ammonium bicarbonate and 0.05% β-mercaptoethanol; both purchased from Sigma Aldrich, Zwijndrecht, The Netherlands; pH 8.2) was added to each well using a pipetting robot (ThermoFisher Multidrop). Afterwards, the plates were incubated for 10 min at 95 °C. Then, the plates were cooled to room temperature followed by the addition of 10 µL of alkylating agent (100 mM iodoacetamide; Sigma Aldrich, Zwijndrecht, The Netherlands) using the same robotic pipet. Subsequently, the plates were

incubated in the dark at room temperature for 30 min. Then, 10 µL of 0.01 µg/µL of trypsin (Promega Benelux B.V. Leiden, The Netherlands) in 50 mM acetic acid was pipetted into each well using the pipetting robot and the plates were incubated overnight at 37 °C. Then, the plates were centrifuged at 1000 rpm for 1 min in an Eppendorf Centrifuge 5810 R. Using the Multidrop. Finally, 10 µL of FA (Formic acid; 1.25%; Biosolve, Valkenswaard, The Netherlands) was added to each well to quench the digestion. The plates were then stored at -20 °C until analysis.

The tryptic digests were analysed using nanoLC-MS/MS according to Slagboom *et al* [22]. From each well, 1 µL was injected by an UltiMate 3000 RSLCnano injector system (Thermo Fisher Scientific, Ermelo, The Netherlands) for subsequent separation. The separation was done on an Acclaim™ PepMap™ 100 C18 HPLC column (150 mm x 75 µm) with a particle size of 2 µm and a pore size of 100-Å in combination with an Acclaim™ PepMap™ 100 C18 trapping column (5 mm x 0.3 mm), with a particle size of 5 µm and a pore size of 100-Å, (Thermo Fisher Scientific, Ermelo, The Netherland). The gradients used were 14.4 minutes long, and the temperature of the column was maintained at 45 °C. Mobile phase A consisted of 98% MQ, 2% ACN and 0.1%, and mobile phase B consisted of 98% ACN, 2% MQ and 0.1% FA. The gradient used for separation started with 1% mobile phase B for 3 min, followed by a linear increase of B to 40% in 7.5 min, followed by a linear increase to 85% in 0.1 min. Next an isocratic elution at 85% for 0.7 min, followed by a linear decrease to 1% mobile phase B in 0.2 min. Finally, the column was equilibrated for 3.7 min at 1% mobile phase B. The mass spectrometer used for detection was a MaXis II QTOF mass spectrometer (Bruker Daltonics, Billerica, Massachusetts, United States of America), which was equipped with a Bruker Captivespray source operating in positive-ion mode. The following ESI source parameters were used: i) capillary voltage 1.6 kV, ii) nanoBooster pressure at 0.20 Bar, iii) source temperature 150°C, and iv) dry gas flow 3.0 L/min. As mass analyser parameters, the following settings were chosen: i)

mass range m/z 50–3000 range, ii) in-source collision induced dissociation (isCID) energy transfer 10 eV, and iii) store rate of 2 Hz. The mass spectrometer was controlled by Bruker Compass software.

S3.1 Processing of High Throughput venomics data

First, MGF files were generated using the ProcessWithMethod function within the Bruker DataAnalysis software. The derived MGF files were then processed using the Mascot Daemon software to convert all single files into one batch. Therefore, the following search parameters were used: i) the instrument type was ESI-q-TOF, ii) the digestion enzyme used was semiTrypsin, iii) allowing one missed cleavage, iv) carbamidomethyl on cysteine was chosen as a fixed modification, v) as variable modifications 2 options were chosen; amidation of protein C-terminus and oxidation on methionine, vi) fragment mass tolerance; ± 0.05 Da vii) ± 0.2 Da peptide for mass tolerance. Subsequently, the extracted information obtained from this process was merged using in-house written R-scripts mentioned in [22]. This processed data was converted to a single Excel file for each venom analysed. This resulted in an excel file containing venomics information on the toxins retrieved for each well from that 384-well plate. The outputted information was: i) protein accession, ii) protein score, iii) theoretical protein mass, iv) protein sequence coverage, v) protein description, vi) full protein sequence and vii) the sequences of the found peptides. Finally, the so-called protein score chromatograms (PSCs) were plotted for each of the two pooled venoms analysed by HT venomics. These PSCs are produced by plotting all protein scores, which was a value obtained by the Mascot software that is correlated to the confidence of the match, from each of the identified toxins on the y-axis versus the retention times of the corresponding wells they were present in on the x-axis. This procedure ends up creating reconstructed protein score peaks for all the toxins retrieved by the venomics procedure, as their protein scores are positively correlated to their concentration in each of the wells.
